# The association between physical activity and health-related quality of life among breast cancer survivors

**DOI:** 10.1186/s12955-017-0706-9

**Published:** 2017-06-30

**Authors:** Woo-kyoung Shin, Sihan Song, So-Youn Jung, Eunsook Lee, Zisun Kim, Hyeong-Gon Moon, Dong-Young Noh, Jung Eun Lee

**Affiliations:** 1 Research Institute of Human Ecology, Seoul National University, Seoul, 08826 South Korea; 2 Department of Food and Nutrition, Sookmyung Women’s University, Seoul, 04310 South Korea; 3 Breast Cancer Center, National Cancer Center, Goyang, 10408 South Korea; 4 Department of Surgery, Soonchunhyang University Bucheon Hospital, Bucheon, 14584 South Korea; 5 Department of Surgery and Cancer Research Institute, Seoul National University College of Medicine, Seoul, 03080 South Korea; 6 Department of Food and Nutrition, Seoul National University, Seoul, 08826 South Korea

**Keywords:** Physical activity, Health related quality of life, Breast cancer survivor, Korean women

## Abstract

**Background:**

The quality of life for breast cancer survivors has become increasingly important because of their high survival rate and prolonged life expectancy. The purpose of this study was to examine the association of physical activity following diagnosis and health-related quality of life (HRQOL) in breast cancer survivors.

**Methods:**

We conducted a cross-sectional study of breast cancer survivors. A total of 231 women aged 21–78 years who had been diagnosed with stages I to III breast cancer and had breast cancer surgery at least 6 months prior were recruited from three hospitals between September 2012 and April 2015 and were included in this study. We asked participants about their HRQOL and engagement in physical activity using structured questionnaires. We examined the association between HRQOL levels and physical activity using a generalized linear model.

**Results:**

Breast cancer survivors in the high physical activity group (3rd tertile) were more likely to have lower scores for fatigue (p for trend = 0.001) and pain (p for trend = 0.02) and higher scores for sexual function (p for trend = 0.007) than those in the low physical activity group (1st tertile). When we stratified participants by stage, we found increasing scores for physical functioning (p for trend =0.01) and decreasing scores for fatigue (p for trend = 0.02) with increasing levels of physical activity in breast cancer survivors with stage I breast cancer. In survivors with stages II and III, we found statistically significant associations with fatigue (p for trend = 0.02) and sexual functioning (p for trend = 0.001).

**Conclusions:**

In conclusion, engagement in physical activity was related to better health-related quality of life among breast cancer survivors. Our findings may warrant further prospective and intervention studies to support the benefit of physical activity in improving the quality of life and survival of Korean breast cancer survivors.

**Electronic supplementary material:**

The online version of this article (doi:10.1186/s12955-017-0706-9) contains supplementary material, which is available to authorized users.

## Background

Breast cancer was the most common cancer among women in the world in 2012 [1]. Exposure to breast cancer risk factors, increases in early detection, and the development of treatment methods have resulted in a substantial increase in the number of breast cancer survivors. The prognosis for Korean women diagnosed with breast cancer has also improved, with 91.3% surviving at least 5 years post-diagnosis in Korea [2].

The majority of breast cancer survivors have mild and moderate levels of physical and psychological treatment side effects, and these can affect their health-related quality of life (HRQOL). Women with breast cancer who received adjuvant chemotherapy tend to experience persistent physical symptoms of cancer and treatment including fatigue [3, 4], pain or sensations in the arm or breast [5], hormone-related symptoms [6, 7], and sexual dysfunction [6, 8]. These symptoms can last for months to even years after completion of cancer treatment and have an adverse effect on their HRQOL [9, 10].

Physical activity is perceived to be an effective non-pharmacological therapy in cancer patients [11, 12] by relieving the distress caused by physical or psychological symptoms. A growing body of evidence supports the idea that increasing physical activity provides important benefits by promoting psychological and physical well-being in cancer patients [12–14]. Recent several studies showed that physical activity had positive effects on physical symptoms, fitness measures, body composition, biological changes such as immune function, psychosocial measures and other multiple aspects of HRQOL [15–21]. However, most of these studies were conducted in Western populations or on only a few survivors in Asia.

Given that research concerning the psychosocial and physical health among breast cancer survivors is important [22], we aimed to examine the association of physical activity following diagnosis and HRQOL in breast cancer survivors.

## Methods

### Study participants

A total of 307 women aged 21 to 78 years were recruited between September 2012 and November 2015. These women had been diagnosed at three large hospitals in Korea with stage I to III breast cancer according to the American Joint Committee on Cancer (AJCC) criteria and had breast cancer surgery at least 6 months before the baseline. We excluded women with missing medical record (*n* = 19), metastasis after breast cancer diagnosis (*n* = 17), other cancers prior to diagnosis (*n* = 6), or any cancer after diagnosis, but before enrollment (*n* = 10). Women were also excluded if they had implausible levels of physical activity, which corresponded to over the top 1% (*n* = 2). As a result, our study included a sample of 231 breast cancer survivors.

All procedures for this study were approved by the institutional review board of each hospital. Written informed consent was obtained from all participants.

### Assessment of physical activity levels

Physical activity after breast cancer diagnosis was assessed using a detailed questionnaire. We asked participants about the type, duration and frequency of each physical activity. As additional questions, participants were asked to list up to three types of exercise that they commonly engaged in as well as their duration and frequency. A metabolic equivalent (MET) value was assigned to each activity reported according to the Compendium of Physical Activities [23].

### Assessment of health-related quality of life

We used a Korean version of the European Organization for Research and Treatment of Cancer (EORTC)**,** Quality of Life Questionnaire Core 30 (QLQ-C30) version 3.0 and Quality of Life Questionnaire Breast Cancer Module 23 (QLQ-BR23), both of which have been validated for Koreans after obtaining written permission from the EORTC Study Group [24, 25].

The EORTC QLQ-C30 is a 30-item core-cancer specific questionnaire-integrating system for assessing the HRQOL of cancer patients participating in international clinical trials [26]. The questionnaire incorporates five functional scales (physical, role, emotional, cognitive, and social), three symptom scales (fatigue, nausea and vomiting, and pain), a global health and QOL scale, and single items for the assessment of additional symptoms commonly reported by cancer patients (e.g., dyspnea, insomnia, appetite loss, constipation, and diarrhea), as well as the perceived financial impact of the disease and treatment [23]. All items are scored on 4-point Likert scales, ranging from 1 (“not at all”) to 4 (“very much”), with the exception of two items in the global health/QOL scale which use modified 7-point linear analog scales [26].

The EORTC QLQ-BR23 is a 23-item breast-cancer-specific questionnaire measuring QOL in breast cancer patients. It incorporates four functional scales (body image, sexual functioning, sexual enjoyment, and future perspective) and four symptom scales (systematic therapy side effects, breast symptoms, arm symptoms, and being upset by hair loss). All items are scored on 4-point Likert scales, ranging from 1 (“not at all”) to 4 (“very much”) [27, 28].

All of the scores from 1 to 4 or from 1 to 7 were converted to a score from 0 to 100 according to the EORTC Scoring manual [26]. A high score for a functional scale represented a high/healthy level of function, a high score for the global health status/QOL represented a high QOL, but a high score for a symptom scale/item represented a high level of symptomatology/problems. A higher score represented a higher (“better”) level of function, or a higher (“worse”) level of symptoms.

### Assessment of demographic, clinical and other lifestyle factors

The demographic questionnaire captured survivors’ dietary supplement use, educational level, marital status, and alcohol intake. We obtained information on survivors’ age at diagnosis, height and weight at diagnosis, survival time, AJCC stage at diagnosis, and menopausal status at diagnosis through medical records. We calculated total energy intake based on information on the foods and amounts consumed using 3-day dietary records for each participant. The participants were asked to write down every food and dish they consumed on 3 non-consecutive days, including 2 weekdays and 1 weekend day.

### Statistical analyses

Categorical data were described as proportions and percentages, and continuous data were described as the mean and standard deviation (SD) for descriptive analysis of characteristics of participants. To compare characteristics, analysis of variance was used for continuous variables, and chi-squared tests for categorical variables. We examined the association between HRQOL level and physical activity using the generalized linear model. The HRQOL level was log-transformed to improve the normality and exponentiated the least square (LS) means. Physical activity levels were grouped into tertiles. Multivariate models included age at diagnosis (years, continuous), energy intake (kcal/day, continuous), dietary supplement use (yes, no), time since surgery (6 months-1 year, 1–5 years, and ≥ 5 years), AJCC stage at diagnosis (stage I/II/III), education level (high school of less/college or more) , marital status (married or cohabitating/unmarried, divorced or widowed), and the hospital where participants were treated. Statistical significance of interaction terms was estimated by the Wald test, by including a cross-product term of the exposure in the generalized linear model. If we had missing variables of energy intake (*n* = 26), alcohol intake (*n* = 1), education level (*n* = 3), and marital status (*n* = 1), we assigned participants to median or the common category. The statistical software package SAS for Windows version 9.4 was used for all statistical analyses. Statistical significance was determined based on *P* values (< 0.05) and 95% confidence intervals.

## Results

A total of 231 survivors, aged 21 to 78 years, with stage I, II, or III breast cancer were included in the analysis. Table 1 presents demographic and clinical characteristics according to physical activity levels. Breast cancer survivors of our study had average of 33.66 MET-hours per week. The mean age of the patients was 48.07 years. More than 60% of the survivors were dietary supplement users (65.37%), postmenopausal at diagnosis (64.50%) and married or cohabiting (81.74%). Approximately 74% of the survivors were enrolled 1 to 5 years after their surgery and over half of the survivors reported ever drinking alcohol. We did not find significant differences in age, BMI, energy intake, dietary supplement use, time since surgery, AJCC stage, menopausal status, alcohol intake, education level, and marital status according to physical activity levels in breast cancer survivors included in this analysis.

**Table 1:** Characteristics of study participants according to physical activity levels

	Physical Activity (MET-hours per week)				
	All (*n* = 231)	Tertile 1 (*n* = 77)	Tertile 2 (*n* = 77)	Tertile 3 (*n* = 77)	P value^a)^
Physical activity (METs-hour per week)^b^	33.66 (29.86)	7.19 (5.29)	26.96 (6.43)	66.83 (27.56)	<0.001
Age (year)^b^	48.07 (8.36)	46.94 (7.91)	49.16 (8.89)	48.12 (8.22)	0.31
Body Mass Index (kg/m^2^)^b^	23.20 (3.13)	23.62 (3.86)	23.07 (2.97)	22.92 (2.38)	0.55
Energy intake (kcal/day)^b^	1778.30 (402.76)	1693.07 (363.22)	1805.71 (398.39)	1825.96 (432.12)	0.16
Dietary supplement use^b^					
yes	151 (65.37)	48 (62.34)	55 (71.43)	48 (62.34)	0.39
no	80 (34.63)	29 (37.66)	22 (28.57)	29 (37.66)	
Time since surgery^b^					
6 month - 1 year	32 (13.85)	12 (15.58)	8 (10.39)	12 (15.58)	0.70
1 year - 5 years	172 (74.46)	58 (75.32)	60 (77.92)	54 (70.13)	
5 years ≤	27 (11.69)	7 (9.09)	9 (11.69)	11 (14.29)	
AJCC stage^b^					
I	101 (43.72)	32 (41.56)	36 (46.75)	33 (42.86)	0.90
II	108 (46.75)	38 (49.35)	35 (45.45)	35 (45.45)	
III	22 (9.52)	7 (9.09)	6 (7.79)	9 (11.69)	
Menopausal status at the diagnosis^b^					
Postmenopause	149 (64.50)	53 (68.83)	51 (66.23)	45 (58.44)	0.37
Premenopause	82 (35.50)	24 (31.17)	26 (33.77)	32 (41.56)	
Alcohol intake^b^					
Never drinker	112 (48.70)	36 (46.75)	42 (54.55)	34 (44.74)	0.44
Ever drinker	118 (51.30)	41 (53.25)	35 (45.45)	42 (55.26)	
Education level^b^					
High school or less	126 (55.26)	38 (49.35)	41 (53.95)	47 (62.67)	0.25
College or more	102 (44.74)	39 (50.65)	35 (46.05)	28 (37.33)	
Marital status^b^					
Married or cohabitation	188 (81.74)	58 (76.32)	61 (79.22)	69 (89.61)	0.08
Unmarried or divorced or widowed	42 (18.26)	18 (23.68)	16 (20.78)	8 (10.39)	

*MET* metabolic equivalent, *AJCC* American Joint Committee on Cancer

^a^Analysis of variance was used for continuous variables and chi-square test was used for categorical variables

^b^Continuous variables are reported as Mean value (sd) and Categorical variables are reported as No. (%)

Increasing physical activity was associated with lower scores of fatigue or pain; LS means (95% CIs) of fatigue for each subsequent tertile were 21.63, 21.00, and 13.30, respectively (p for trend = 0.001) (Table 2). For pain, LS means (95% CIs) for each subsequent tertile were 12.45, 7.9, and 6.25, respectively (p for trend = 0.02). Breast cancer survivors in the high physical activity group (3rd tertile) were more likely to have higher scores of sexual functioning (p for trend =0.007), than those in the low physical activity group (1st tertile). When we stratified participants by stage, we found increasing scores of physical functioning (p for trend =0.01) and decreasing scores of fatigue (p for trend =0.02) with increasing levels of physical activity in breast cancer survivors with stage I (Table 3). Among breast cancer survivors with stage II and III, we found lower scores of fatigue (p for trend = 0.02), but higher scores of sexual functioning (p for trend = 0.001) comparing the 3rd tertile with the 1st tertitle of physical activity levels (Table 4).

**Table 2:** Health-related quality of life (HRQOL) scores according to physical activity levels among breast cancer survivors with stage I to III breast cancer

HRQOL Items	Physical Activity (MET-hours per week)				
	All	Tertile 1	Tertile 2	Tertile 3	P for trend^a^
EORTC QLQ-C30, LS means (95% CI)^b^					
Global health status / QoL	206	45.25 (33.62, 60.90)	31.99 (23.14, 44.22)	40.10 (29.17, 55.12)	0.64
Functioning					
Physical Functioning	229	78.44 (70.20, 87.65)	81.12 (71.93, 91.48)	82.53 (73.21, 93.05)	0.41
Role Functioning	230	75.68 (59.77, 95.82)	83.78 (64.68, 108.51)	90.10 (69.98, 116.01)	0.18
Emotional Functioning	231	75.16 (62.37, 90.58)	72.87 (59.47, 89.31)	75.67 (61.95, 92.44)	0.89
Cognitive Functioning	231	75.13 (63.80, 88.47)	72.40 (60.60, 86.51)	71.47 (59.98, 85.16)	0.59
Social Functioning	231	56.74 (44.62, 72.15)	73.08 (56.25, 94.94)	72.96 (56.39, 94.39)	0.08
Symptom					
Fatigue	230	21.63 (16.07, 29.11)	21.00 (15.20, 29.02)	13.30 (9.64, 18.36)	0.001
Nausea / Vomiting	231	2.56 (1.60, 4.09)	2.33 (1.40, 3.89)	2.54 (1.53, 4.20)	0.97
Pain	230	12.45 (7.44, 20.83)	7.90 (4.51, 13.83)	6.25 (3.58, 10.93)	0.02
Dyspnea	228	5.83 (3.38, 10.03)	3.23 (1.79, 5.85)	4.07 (2.25, 7.37)	0.35
Insomnia	229	12.60 (7.38, 21.53)	15.79 (8.81, 28.29)	16.22 (9.07, 29.00)	0.42
Appetite loss	228	2.22 (1.33, 3.70)	2.69 (1.54, 4.69)	2.10 (1.21, 3.65)	0.72
Constipation	229	3.69 (2.09, 6.52)	4.36 (2.35, 8.09)	3.62 (1.95, 6.69)	0.86
Diarrhea	231	2.95 (1.77, 4.91)	2.26 (1.30, 3.95)	2.35 (1.36, 4.07)	0.48
Financial Problems	231	4.46 (2.56, 7.77)	5.96 (3.26, 10.90)	3.29 (1.81, 5.95)	0.19
EORTC QLQ-BR23, LS means (95% CI)^b^					
Functioning					
Body image	231	36.03 (24.05, 54.00)	34.95 (22.49, 54.30)	32.14 (20.83, 49.58)	0.58
Sexual functioning	209	2.69 (1.53, 4.71)	2.57 (1.43, 4.65)	5.34 (3.04, 9.39)	0.007
Sexual enjoyment	69	16.89 (4.80, 59.47)	9.65 (3.14, 29.62)	11.54 (3.34, 39.90)	0.56
Future perspective	231	17.83 (10.65, 29.83)	18.04 (10.29, 31.60)	19.72 (11.35, 34.24)	0.70
Symptom					
Systematic therapy side effects	231	21.59 (16.54, 28.19)	23.55 (17.62, 31.49)	19.06 (14.32, 25.36)	0.29
Breast symptoms	231	12.45 (8.04, 19.28)	10.06 (6.25, 16.20)	10.42 (6.52, 16.65)	0.52
Arm symptoms	231	18.20 (12.41, 26.69)	21.04 (13.86, 31.93)	17.92 (11.89, 27.02)	0.83
Upset by hair loss	157	17.28 (8.92, 33.50)	14.78 (7.09, 30.83)	17.59 (8.60, 36.00)	0.90

*MET* metabolic equivalent, *EORTC QLQ-C30* European organization for research and treatment of cancer quality of life questionnaire core 30, *LS* least-squares, *CI* confidence interval, *EORTC QLQ-BR23* European organization for research and treatment of cancer quality of life questionnaire breast cancer module 23

^a^P for trend was calculated using the median value of each tertile category as a continuous variable

^b^Adjusted for age (year: continuous), energy intake (kcal/day: continuous), dietary supplement use (yes, no), education level (high school or less, college or more), marital status (married or cohabitated, unmarried or divorced or widowed), time since surgery (6 month – 1 year, 1 year - 5 years, ≥5 years), stage (I, II, or III), and center

**Table 3:** Health-related quality of life (HRQOL) scores according to physical activity levels among breast cancer survivors with stage I breast cancer

HRQOL Items	Physical Activity (MET-hours per week)				
	All	Tertile 1	Tertile 2	Tertile 3	P for trend^a^
EORTC QLQ-C30, LS means (95% CI)^b^					
Global health status / QoL	93	44.40 (27.04, 72.90)	33.27 (20.51, 53.98)	34.72 (20.75, 58.08)	0.35
Functioning					
Physical Functioning	101	74.97 (67.56, 83.20)	76.85 (69.59, 84.87)	84.62 (76.17, 94.00)	0.01
Role Functioning	101	79.69 (60.00, 105.84)	71.11 (54.25, 93.20)	88.10 (66.14, 117.35)	0.36
Emotional Functioning	101	83.73 (57.88, 121.12)	79.85 (56.16, 113.54)	80.11 (55.17, 116.33)	0.83
Cognitive Functioning	101	77.91 (65.43, 92.78)	77.49 (65.61, 91.52)	67.46 (56.55, 80.48)	0.07
Social Functioning	101	57.30 (39.62, 82.86)	70.40 (49.53, 100.07)	76.72 (52.85, 111.36)	0.12
Symptom					
Fatigue	101	27.95 (17.23, 45.34)	25.32 (15.97, 40.16)	16.29 (9.99, 26.55)	0.02
Nausea / Vomiting	101	2.56 (1.15, 5.70)	2.75 (1.28, 5.89)	3.06 (1.37, 6.87)	0.64
Pain	101	11.16 (4.92, 25.29)	7.64 (3.50, 16.65)	5.92 (2.59, 13.52)	0.12
Dyspnea	99	5.25 (2.13, 12.96)	3.46 (1.46, 8.21)	4.02 (1.60, 10.13)	0.63
Insomnia	101	14.61 (6.19, 34.49)	14.90 (6.57, 33.78)	22.14 (9.30, 52.72)	0.29
Appetite loss	100	1.35 (0.54, 3.38)	2.04 (0.86, 4.84)	1.57 (0.63, 3.91)	0.85
Constipation	101	3.70 (1.42, 9.67)	6.01 (2.41, 15.02)	8.35 (3.17, 22.04)	0.09
Diarrhea	101	3.01 (1.36, 6.67)	2.27 (1.06, 4.84)	1.63 (0.73, 3.65)	0.12
Financial Problems	101	2.82 (1.12, 7.07)	3.20 (1.33, 7.69)	1.92 (0.76, 4.86)	0.33
EORTC QLQ-BR23, LS means (95% CI)^b^					
Functioning					
Body image	101	68.77 (39.53, 119.62)	65.36 (38.56, 110.80)	57.80 (33.04, 101.11)	0.51
Sexual functioning	94	4.12 (1.54, 11.03)	4.56 (1.80, 11.54)	5.57 (2.12, 14.60)	0.50
Sexual enjoyment	33	53.86 (7.89, 367.75)	36.75 (7.85, 172.11)	21.84 (3.57, 133.78)	0.25
Future perspective	101	25.27 (10.25, 62.28)	30.21 (12.79, 71.39)	29.68 (11.93, 73.83)	0.75
Symptom					
Systematic therapy side effects	101	16.48 (10.39, 26.14)	15.77 (10.16, 24.48)	12.99 (8.15, 20.70)	0.27
Breast symptoms	101	5.94 (3.08, 11.44)	6.82 (3.65, 12.74)	5.27 (2.72, 10.22)	0.63
Arm symptoms	101	13.47 (7.64, 23.77)	13.75 (8.00, 23.62)	11.58 (6.52, 20.54)	0.55
Upset by hair loss	69	14.10 (4.83, 41.22)	7.35 (2.41, 22.41)	15.16 (4.30, 53.40)	0.80

*MET* metabolic equivalent, *EORTC QLQ-C30* European organization for research and treatment of cancer quality of life questionnaire core 30, *LS* least-squares, *CI* confidence interval, *EORTC QLQ-BR23* European organization for research and treatment of cancer quality of life questionnaire Breast cancer module 23

^a^P for trend was calculated using the median value of each tertile category as a continuous variable

^b^Adjusted for age (year: continuous), energy intake (kcal/day: continuous), dietary supplement use (yes, no), education level (high school or less, college or more), marital status (married or cohabitated, unmarried or divorced or widowed), time since surgery (6 month – 1 year, 1 year - 5 years, ≥5 years), and center

**Table 4:** Health-related quality of life (HRQOL) scores according to physical activity levels among breast cancer survivors with stage II and III breast cancer

HRQOL Items	Physical Activity (MET-hours per week)				
	All	Tertile 1	Tertile 2	Tertile 3	P for trend^a^
EORTC QLQ-C30, LS means (95% CI)^b^					
Global health status / QoL	113	48.33 (32.27, 72.39)	31.37 (19.83, 49.61)	43.56 (28.54, 66.49)	0.89
Functioning					
Physical Functioning	128	81.46 (68.05, 97.51)	85.19 (69.31, 104.72)	81.99 (67.23, 99.98)	0.98
Role Functioning	129	76.34 (53.02, 109.91)	100.65 (65.83, 153.89)	95.71 (64.41, 142.23)	0.36
Emotional Functioning	130	72.94 (59.76, 89.03)	67.19 (53.42, 84.51)	75.44 (60.73, 93.71)	0.63
Cognitive Functioning	130	77.05 (59.64, 99.56)	69.47 (51.73, 93.29)	75.00 (56.74, 99.13)	0.96
Social Functioning	130	59.44 (42.46, 83.22)	78.02 (52.98, 114.91)	70.92 (49.17, 102.29)	0.49
Symptom					
Fatigue	129	18.19 (12.29, 26.91)	19.25 (12.25, 30.25)	11.47 (7.43, 17.71)	0.02
Nausea / Vomiting	130	2.53 (1.38, 4.63)	2.20 (1.09, 4.41)	2.17 (1.12, 4.19)	0.69
Pain	129	11.40 (5.74, 22.67)	7.32 (3.32, 16.17)	6.15 (2.87, 13.17)	0.14
Dyspnea	129	5.29 (2.60, 10.74)	2.90 (1.28, 6.57)	3.93 (1.79, 8.61)	0.63
Insomnia	128	12.16 (6.05, 24.45)	19.57 (8.75, 43.75)	12.05 (5.56, 26.12)	0.78
Appetite loss	128	2.28 (1.25, 4.15)	3.06 (1.53, 6.09)	2.11 (1.09, 4.10)	0.67
Constipation	128	3.90 (1.87, 8.15)	3.56 (1.53, 8.27)	1.93 (0.86, 4.34)	0.08
Diarrhea	130	2.72 (1.36, 5.45)	2.11 (0.95, 4.69)	3.51 (1.65, 7.46)	0.40
Financial Problems	130	5.33 (2.56, 11.10)	8.65 (3.72, 20.11)	4.30 (1.94, 9.56)	0.42
EORTC QLQ-BR23, LS means (95% CI)^b^					
Functioning					
Body image	130	26.17 (14.74, 46.48)	24.74 (12.78, 47.91)	22.67 (12.13, 42.35)	0.66
Sexual functioning	115	1.97 (1.00, 3.85)	1.61 (0.76, 3.41)	5.77 (2.92, 11.39)	0.001
Sexual enjoyment	36	10.24 (1.60, 65.60)	3.36 (0.64, 17.61)	7.46 (1.29, 43.12)	0.97
Future perspective	130	16.23 (8.49, 31.02)	12.63 (5.99, 26.62)	15.43 (7.62, 31.23)	>0.99
Symptom					
Systematic therapy side effects	130	23.04 (16.92, 31.36)	30.31 (21.25, 43.22)	23.11 (16.52, 32.33)	0.75
Breast symptoms	130	16.84 (9.26, 30.60)	11.09 (5.58, 22.06)	14.16 (7.39, 27.13)	0.77
Arm symptoms	130	19.83 (11.69, 33.65)	26.64 (14.50, 48.95)	22.71 (12.77, 40.37)	0.78
Upset by hair loss	88	19.92 (7.97, 49.81)	26.82 (9.60, 74.98)	22.28 (8.72, 56.92)	0.89

*MET* metabolic equivalent, *EORTC QLQ-C30* European organization for research and treatment of cancer quality of life questionnaire core 30, *LS* least-squares, *CI* confidence interval, *EORTC QLQ-BR23* European organization for research and treatment of cancer quality of life questionnaire breast cancer module 23

^a^P for trend was calculated using the median value of each tertile category as a continuous variable

^b^Adjusted for age (year: continuous), energy intake (kcal/day: continuous), dietary supplement use (yes, no), education level (high school or less, college or more), marital status (married or cohabitated, unmarried or divorced or widowed), time since surgery (6 month – 1 year, 1 year - 5 years, ≥ 5 years), stage (II or III), and center

We further examined whether BMI, menopausal status at diagnosis, and time since surgery modified the associations of fatigue, pain, and sexual functioning, all of which reached significance in the main analysis. Scores of fatigue decreased with increasing levels of physical activity in both strata of BMI (*p* values for trend = 0.03 for BMI < 23 kg/m^2^ and 0.01 for BMI ≥ 23 kg/m^2^) (Additional file 1: Table S1). Significant trends were observed for pain among survivors with BMI ≥ 23 kg/m^2^ and for sexual functioning among survivors with BMI < 23 kg/m^2^, but these interactions were not statistically significant.

Menopausal status at diagnosis did not modify the associations of fatigue, pain and sexual functioning with physical activity (Additional file 1: Table S2). We observed decreasing scores of fatigue with increasing levels of physical activity for both pre- and post-menopausal breast cancer survivors. Although the interaction was not statistically significant, decreasing trends of pain and increasing trends of sexual functioning with increasing levels of physical activity were limited to post-menopausal breast cancer survivors.

We found that fatigue was associated with physical activity levels regardless of time since surgery (Additional file 1: Table S3). Although a decreasing trend for pain and increasing trend for sexual functioning were more evident among survivors who had surgery less than 2 years before the study compared to those who had surgery 2 or more years before the study, these interactions were not statistically significant.

We examined whether age at diagnosis (<48, ≥48 years, median) modified the associations for fatigue, pain, and sexual functioning (Additional file 1: Table S4). We found that decreasing scores of fatigue with increasing levels of physical activity for both age groups, but these interactions were not statistically significant. Significant trend for pain was limited to breast cancer survivors with ≥48 years of age at diagnosis, but to those with <48 years of age at diagnosis for sexual functioning, albeit statistically not significant.

## Discussion

We aimed to determine whether physical activity levels after breast cancer diagnosis were related to HRQOL, and these associations varied by stage, BMI, menopausal status at diagnosis, time since surgery, and age at diagnosis among breast cancer survivors in Korea. We found that higher physical activity after diagnosis was associated with lower scores of fatigue and pain and higher scores of sexual functioning. When we limited our analysis to stage I or stage II/III, an inverse association for fatigue remained statistically significant in both groups, but physical functioning increased only among survivors with stage I cancer and sexual functioning increased only among survivors with stage II or III cancer, with increasing levels of physical activity.

Consistent with our research, several observational studies found that exercise was linked to improvement of HRQOL. In a prospective study of the Health Eating Activity and Lifestyle (HEAL) study of breast cancer prognosis, pre- and post-diagnosis recreational physical activity was associated with better physical functioning, and increases in physical activity after cancer diagnosis were associated with less fatigue and pain and better physical functioning in 545 breast cancer survivors [29]. In a large prospective cohort study of breast cancer survivors conducted in Shanghai, women with higher exercise MET scores (≥8.3 MET-hours per week) were more likely to have higher scores of total QOL, and the exercise-QOL association remained stable overtime after cancer diagnosis [30]. Other observational studies in Norway [31], Italy [32], the USA [33, 34], and Finland [35] also found that physical activity was associated with improved QOL or less fatigue.

Recent meta-analyses of intervention studies reported that exercise reduced fatigue [11, 36] and QOL [36] among breast cancer survivors. Recent large randomized clinical trials found that exercise intervention improved cancer survivors’ QOL. The Better Exercise Adherence after Treatment for Cancer (BEAT Cancer) study randomized 222 breast cancer survivors who finished primary treatment to a 3-month combined exercise program or usual care and found better QOL at 3 and 6 months in the intervention group than in the usual care group [37]. The Exercise and Nutrition Enhance Recovery and Good Health for You (ENERGY) reported that intensive group-based intervention of diet and physical activity compared to non-intensive attention control improved physical function of HRQOL at 6 and 12 months of intervention, but the difference between intervention and control groups diminished at 24 months [38]. Likewise, a 12-week LIVESTRONG exercise program at the YMCA led to improvement of QOL in 186 cancer survivors (52% were breast cancer survivors) [39].

Small intervention studies of 40 to 59 Korean breast cancer survivors also reported that exercise was beneficial for the reduction of fatigue [40, 41] and appetite loss [42] and increases in emotional [41] and physical function [42]. A 12-week mobile application-based exercise intervention improved HRQOL in Korean breast cancer patients [43]. In a randomized trial of 277 breast and colorectal cancer patients (168 with breast cancer), the provision of an exercise motivation package including exercise DVDs, a pedometer, an exercise diary, and an exercise education session increased the role of physical function and decreased diarrhea [44]. A pretest–posttest intervention study suggested that the intervention group with print materials and pedometers showed significantly improved QOL and reduced fatigue compared to standard recommendation group [45].

Several studies have reported that exercise therapy for cancer patients during radiotherapy reduced fatigue, but the reason is not clear. An intervention study of 66 male prostate cancer patients suggested that improvements in physical function by exercise therapy may help overcome radiation fatigue [46]. There is scientific evidence that exercise training can reduce fatigue and improve the QOL of cancer patients and survivors [47]. Doing exercise on cancer patients or survivors could improve in their functional capacity and increase tolerance to physical fatigue and metabolic efficiency [48]. Improved metabolic efficiency can change characteristics of skeletal-muscle, and increase the proportion of oxidative fibers or decrease the proportion of glycolytic fibers [49]. Oxidative fibers can remove lactate from blood and they are less fatigable. Therefore, increased muscle efficiency explains how patients with higher physical activity can carry out normal daily activities with less fatigue [47].

Our study had several limitations. First, our study design did not enable us to determine casual direction because we did not measure HRQOL levels after physical activity assessment. Further prospective studies are needed to evaluate changes in HRQOL over time according to physical activity. Secondly, we did not have information on pre-diagnostic levels of physical activity, and our sample size was small. However, given that no prospective cohort studies have been conducted on physical activity and HRQOL or mortality in Korea, to our knowledge, this study provides evidence demonstrating the importance of physical activity on Korean breast cancer survivors. Thirdly, we cannot rule out that possibility of information bias by interviewer or interviewee because both physical activity and HRQOL were assessed at the same time. Also, misclassification of physical activity or HRQOL could be introduced. However, given that MET-hour per week of physical activity was well-correlated to physical functioning of quality of life, ranking of physical activity levels might be captured in our study. Lastly, the sampling for this study was not random, which limits our ability to generalize our results to all breast cancer survivors in Korea. However, because the hospitals in our study are major hospitals in metro areas, attracting patients from all over the country, our results may not be confined to narrow scope of subjects. Also, the positive association between physical activity and better quality of life that we observed may not be limited to our study population because of its potential biological and psychological basis.

## Conclusion

In conclusion, engagement in physical activity was related to less fatigue and pain and better sexual functioning among Korean breast cancer survivors. Our findings may warrant further prospective and intervention studies to support the benefit of physical activity in improving the quality of life and survival of Korean breast cancer survivors.

## Additional file

Additional file 1: Table S1. Health-related quality of life (HRQOL) scores according to physical activity levels among breast cancer survivors by body mass index. **Table S2.** Health-related quality of life (HRQOL) scores according to physical activity levels among breast cancer survivors by menopausal status at the diagnosis. **Table S3.** Health-related quality of life (HRQOL) scores according to physical activity levels among breast cancer survivors by time since surgery. **Table S4.** Health-related quality of life (HRQOL) scores according to physical activity levels among breast cancer survivors by age at diagnosis. (DOCX 48 kb)

## Abbreviations

AJCC: American Joint Committee on Cancer
BEAT Cancer: Better Exercise Adherence after Treatment for Cancer
ENERGY: Exercise and Nutrition Enhance Recovery and Good Health for You
EORTC: European Organization for Research and Treatment of Cancer
HEAL: Health Eating Activity and Lifestyle
HRQOL: Health-related quality of life
LS: Least square
MET: Metabolic equivalent
QLQ-BR23: Quality of Life Questionnaire Breast Cancer Module 23
QLQ-C30: Quality of Life Questionnaire Core 30
