# Supplementary material for: The association between physical activity and health-related quality of life among breast cancer survivors
Source: Health Qual Life Outcomes. 2017 Jun 30;15:132. doi: 10.1186/s12955-017-0706-9 (PMC5493872; doi:10.1186/s12955-017-0706-9)
Supplement: Additional file 1: Table S1. — Health-related quality of life (HRQOL) scores according to physical activity levels among breast cancer survivors by body mass index. Table S2. Health-related quality of life (HRQOL) scores according to physical activity levels among breast cancer survivors by menopausal status at the diagnosis. Table S3. Health-related quality of life (HRQOL) scores according to physical activity levels among breast cancer survivors by time since surgery. Table S4. Health-related quality of life (HRQOL) scores according to physical activity levels among breast cancer survivors by age at diagnosis. (DOCX 48 kb) [file 12955_2017_706_MOESM1_ESM.docx]

Table S1. Health-related quality of life (HRQOL) scores according to physical activity levels among breast cancer survivors by body mass index

|  |  | Physical Activity (MET-hours per week) | | |  |  |
| --- | --- | --- | --- | --- | --- | --- |
| HRQOL Items | All | Tertile 1 | Tertile 2 | Tertile 3 | P for trend^a^ | P for interaction^b^ |
| EORTC QLQ-C30, LS means (95% CI)^c^ |  |  |  |  |  |  |
| Symptom |  |  |  |  |  |  |
| Fatigue |  |  |  |  |  |  |
| BMI < 23 kg/m^2^ | 122 | 27.67 (18.71, 40.94) | 26.73 (17.77, 40.21) | 17.69 (11.89, 26.34) | 0.03 | 0.32 |
| BMI ≥ 23 kg/m^2^ | 108 | 17.04 (10.41, 27.89) | 15.64 (9.02, 27.13) | 9.04 (5.25, 15.57) | 0.01 |  |
| Pain |  |  |  |  |  |  |
| BMI < 23 kg/m^2^ | 122 | 13.44 (6.35, 28.45) | 13.54 (6.20, 29.59) | 8.15 (3.80, 17.46) | 0.18 | 0.51 |
| BMI ≥ 23 kg/m^2^ | 108 | 12.23 (5.61, 26.70) | 4.14 (1.73, 9.91) | 4.63 (1.96, 10.97) | 0.03 |  |
| EORTC QLQ-BR23, LS means (95% CI)^c^ |  |  |  |  |  |  |
| Functioning |  |  |  |  |  |  |
| Sexual functioning |  |  |  |  |  |  |
| BMI < 23 kg/m^2^ | 114 | 1.71 (0.77, 3.80) | 3.78 (1.69, 8.47) | 6.65 (3.03, 14.60) | 0.002 | 0.13 |
| BMI ≥ 23 kg/m^2^ | 95 | 3.02 (1.31, 7.01) | 1.53 (0.61, 3.84) | 4.89 (2.12, 11.27) | 0.14 |  |

*MET* Metabolic Equivalent, *BMI* Body Mass Index, *EORTC QLQ-C30* European Organization for Research and Treatment of Cancer Quality of life Questionnaire Core 30, *LS* Least-squares, *CI* Confidence Interval, *EORTC QLQ-BR23* European Organization for Research and Treatment of Cancer Quality of life Questionnaire Breast Cancer Module 23

^a^P for trend was calculated using the median value of each tertile category as a continuous variable

^b^P for interaction was calculated by the Wald test of cross-product terms

^c^Adjusted for age (year: continuous), energy intake (kcal/day: continuous), dietary supplement use (yes, no), education level (high school or less, college or more), marital status (married or cohabitated, unmarried or divorced or widowed), time since surgery (6 month – 1 year, 1 year - 5 years, ≥5 years), stage (I, II or III), and center

Table S2. Health-related quality of life (HRQOL) scores^1)^ according to physical activity levels among breast cancer survivors by menopausal status at the diagnosis

|  |  | Physical Activity (MET-hours per week) | | |  |  |
| --- | --- | --- | --- | --- | --- | --- |
| HRQOL Items | All | Tertile 1 | Tertile 2 | Tertile 3 | P for trend^a^ | P for interaction^b^ |
| EORTC QLQ-C30, LS means (95% CI)^c^ |  |  |  |  |  |  |
| Symptom |  |  |  |  |  |  |
| Fatigue |  |  |  |  |  |  |
| Premenopausal | 82 | 31.86 (19.63, 51.71) | 21.99 (13.33, 36.29) | 17.56 (11.14, 27.68) | 0.02 | 0.69 |
| Postmenopausal | 148 | 19.93 (13.32, 29.84) | 21.08 (13.40, 33.16) | 13.08 (8.13, 21.04) | 0.04 |  |
| Pain |  |  |  |  |  |  |
| Premenopausal | 82 | 11.36 (4.29, 30.06) | 5.28 (1.93, 14.44) | 6.08 (2.44, 15.19) | 0.27 | 0.85 |
| Postmenopausal | 148 | 14.54 (7.50, 28.20) | 9.58 (4.55, 20.16) | 6.82 (3.12, 14.87) | 0.04 |  |
| EORTC QLQ-BR23, LS means (95% CI)^c^ |  |  |  |  |  |  |
| Functioning |  |  |  |  |  |  |
| Sexual functioning |  |  |  |  |  |  |
| Premenopausal | 77 | 1.21 (0.47, 3.14) | 1.95 (0.72, 5.24) | 2.48 (1.05, 5.87) | 0.13 | 0.92 |
| Postmenopausal | 132 | 3.30 (1.56, 6.99) | 2.72 (1.24, 5.96) | 6.88 (3.14, 15.09) | 0.02 |  |

*MET* Metabolic Equivalent, *EORTC QLQ-C30* European Organization for Research and Treatment of Cancer Quality of life Questionnaire Core 30, *LS* Least-squares, *CI* Confidence Interval, *EORTC QLQ-BR23* European Organization for Research and Treatment of Cancer Quality of life Questionnaire Breast Cancer Module 23

^a^P for trend was calculated using the median value of each tertile category as a continuous variable

^b^P for interaction was calculated by the Wald test of cross-product terms

^c^Adjusted for age (year: continuous), energy intake (kcal/day: continuous), dietary supplement use (yes, no), education level (high school or less, college or more), marital status (married or cohabitated, unmarried or divorced or widowed), time since surgery (6 month – 1 year, 1 year - 5 years, ≥5 years), stage (I, II or III), and center

Table S3. Health-related quality of life (HRQOL) scores^1)^ according to physical activity levels among breast cancer survivors by time since surgery

|  |  | Physical Activity (MET-hours per week) | | |  |  |
| --- | --- | --- | --- | --- | --- | --- |
| HRQOL Items | All | Tertile 1 | Tertile 2 | Tertile 3 | P for trend^a^ | P for interaction^b^ |
| EORTC QLQ-C30, LS means (95% CI)^c^ |  |  |  |  |  |  |
| Symptom |  |  |  |  |  |  |
| Fatigue |  |  |  |  |  |  |
| Time since surgery < 2years | 110 | 27.93 (19.35, 40.31) | 25.60 (16.74, 39.15) | 18.06 (11.61, 28.09) | 0.05 | 0.69 |
| Time since surgery ≥ 2years | 120 | 26.09 (16.99, 40.07) | 27.50 (17.59, 43.01) | 16.15 (10.27, 25.40) | 0.02 |  |
| Pain |  |  |  |  |  |  |
| Time since surgery < 2years | 110 | 20.14 (11.34, 35.79) | 10.60 (5.45, 20.61) | 8.40 (4.21, 16.78) | 0.02 | 0.34 |
| Time since surgery ≥ 2years | 120 | 9.00 (4.13, 19.57) | 8.00 (3.56, 17.98) | 6.11 (2.69, 13.89) | 0.34 |  |
| EORTC QLQ-BR23, LS means (95% CI)^c^ |  |  |  |  |  |  |
| Functioning |  |  |  |  |  |  |
| Sexual functioning |  |  |  |  |  |  |
| Time since surgery < 2years | 99 | 1.85 (0.81, 4.22) | 2.56 (0.99, 6.60) | 5.71 (2.32, 14.07) | 0.01 | 0.09 |
| Time since surgery ≥ 2years | 110 | 3.08 (1.36, 6.95) | 1.99 (0.89, 4.46) | 4.25 (1.96, 9.19) | 0.19 |  |

*MET* Metabolic Equivalent, *EORTC QLQ-C30* European Organization for Research and Treatment of Cancer Quality of life Questionnaire Core 30, *LS* Least-squares, *CI* Confidence Interval, *EORTC QLQ-BR23* European Organization for Research and Treatment of Cancer Quality of life Questionnaire Breast Cancer Module 23

^a^P for trend was calculated using the median value of each tertile category as a continuous variable

^b^P for interaction was calculated by the Wald test of cross-product terms

^c^Adjusted for age (year: continuous), energy intake (kcal/day: continuous), dietary supplement use (yes, no), education level (high school or less, college or more), marital status (married or cohabitated, unmarried or divorced or widowed), time since surgery (6 month – 1 year, 1 year - 5 years, ≥5 years), stage (I, II or III), and center

Table S4. Health-related quality of life (HRQOL) scores^1)^ according to physical activity levels among breast cancer survivors by age at diagnosis

|  |  | Physical Activity (MET-hours per week) | | |  |  |
| --- | --- | --- | --- | --- | --- | --- |
| HRQOL Items | All | Tertile 1 | Tertile 2 | Tertile 3 | P for trend^a^ | P for interaction^b^ |
| EORTC QLQ-C30, LS means (95% CI)^c^ |  |  |  |  |  |  |
| Symptom |  |  |  |  |  |  |
| Fatigue |  |  |  |  |  |  |
| Age < 48 years | 107 | 28.55 (19.41, 41.98) | 26.02 (16.35, 41.39) | 16.81 (10.56, 26.74) | 0.03 | 0.86 |
| Age ≥ 48 years | 123 | 26.83 (17.88, 40.26) | 27.14 (18.48, 39.87) | 15.75 (10.46, 23.71) | 0.02 |  |
| Pain |  |  |  |  |  |  |
| Age < 48 years | 107 | 7.75 (4.03, 14.90) | 8.88 (4.04, 19.49) | 6.76 (3.08, 14.85) | 0.67 | 0.09 |
| Age ≥ 48 years | 123 | 20.74 (10.53, 40.85) | 8.45 (4.44, 16.06) | 5.87 (2.97, 11.63) | 0.003 |  |
| EORTC QLQ-BR23, LS means (95% CI)^c^ |  |  |  |  |  |  |
| Functioning |  |  |  |  |  |  |
| Sexual functioning |  |  |  |  |  |  |
| Age < 48 years | 102 | 2.88 (1.43, 5.79) | 4.48 (1.98, 10.16) | 9.37 (4.14, 21.23) | 0.01 | 0.41 |
| Age ≥ 48 years | 107 | 1.72 (0.72, 4.10) | 1.23 (0.57, 2.65) | 3.03 (1.44, 6.34) | 0.09 |  |

*MET* Metabolic Equivalent, *EORTC QLQ-C30* European Organization for Research and Treatment of Cancer Quality of life Questionnaire Core 30, *LS* Least-squares, *CI* Confidence Interval, *EORTC QLQ-BR23* European Organization for Research and Treatment of Cancer Quality of life Questionnaire Breast Cancer Module 23

^a^P for trend was calculated using the median value of each tertile category as a continuous variable

^b^P for interaction was calculated by the Wald test of cross-product terms

^c^Adjusted for age (year: continuous), energy intake (kcal/day: continuous), dietary supplement use (yes, no), education level (high school or less, college or more), marital status (married or cohabitated, unmarried or divorced or widowed), time since surgery (6 month – 1 year, 1 year - 5 years, ≥5 years), stage (I, II or III), and center
